# Sea buckthorn seed oil triggers mitochondrial dysfunction and apoptosis in *Aspergillus niger* by activating the AGE-RAGE-like signaling pathway

**DOI:** 10.3389/fmicb.2026.1762938

**Published:** 2026-04-21

**Authors:** Yanhua Xin, Aiyu Qin, Jie Yang, Yixin Shen, Jing Li, Lanying Guo, Bin Liang, Jing Su

**Affiliations:** Department of Bioengineering, Xinzhou Normal University, Xinzhou, China

**Keywords:** AGE-RAGE pathway, antifungal mechanism, *Aspergillus niger*, molecular dynamics, network pharmacology, sea buckthorn seed oil, transcriptomics

## Abstract

**Background:**

Fungal infections, in particular those caused by *Aspergillus niger* (*A. niger*), pose a severe threat to human health, and the situation has been getting worse because of increasing resistance against the frontline triazole antifungals. Sea buckthorn (*Hippophae rhamnoides* L.) seed oil (SBT oil) exhibits potent antifungal activity against *A. niger*, principally by disrupting mitochondrial function. Its general mode of action is, nevertheless, unclear.

**Methods:**

We employed an integrated approach combining network pharmacology, transcriptomics, molecular docking, molecular dynamics (MD) simulations, and experimental validation to systematically investigate the antifungal mechanism of SBT oil against *A. niger*.

**Results:**

Network pharmacology identified seven key bioactive compounds and 26 potential targets, with enrichment analysis highlighting the advanced glycation end-products (AGE)–receptor for AGEs (RAGE) signaling pathway as a crucial mechanism. Subsequent transcriptomic and experimental validation in *A. niger* revealed that SBT oil disrupts a functionally analogous stress response pathway, leading to mitochondrial dysfunction and apoptosis. Transcriptomics revealed 580 differentially expressed genes (DEGs), indicating widespread disruption of metabolic networks. Molecular docking and MD simulations confirmed the stable binding of key compounds to core targets, MAPK3 and BCL2. *In vitro* experiments showed that SBT oil increases endogenous AGEs, leading to mitochondrial dysfunction, reduced mitochondrial membrane potential (∆Ψm), elevated ROS and MDA levels, inhibited SDH and ATPase activities, and modulation of MAPK3 and METACASP expression within the AGE-RAGE-like pathway in *A. niger*.

**Conclusion:**

This study provides strong evidence that SBT seed oil exerts its antifungal effects against *A. niger* primarily through amplification of AGE-RAGE-like signaling, offering a solid foundation for its development as a natural antifungal agent.

## 1 Introduction

The increasing prevalence of fungal infections worldwide, especially among immunocompromised populations, presents a considerable challenge to public health (Suleyman and Alangaden, 2021). *Aspergillus niger* (*A. niger*) is recognized as the second most prevalent causative agent of opportunistic fungal infections, following *Candida* species (Pawar and Thaker, 2006). *A. niger*, in addition to its clinical implications, serves as a common spoilage organism, affecting food, plants, and medicinal herbs, which results in significant economic losses (Sebti et al., 2010). *A. niger* can cause severe respiratory diseases, such as pneumonia (Njunda et al., 2012; Oda et al., 2013), and has been identified as the leading causative agent of otomycosis among especially susceptible groups of patients, including people with hematological malignancies or those with a weakened immune system (Chappe et al., 2018). Triazole antifungals are the first-line treatment, but their efficacy is often compromised by host factors and increasing global resistance to antimicrobial drugs (Nywening et al., 2020). The rapid development of triazole resistance in *A. niger* creates an urgent need to develop novel antifungal agents with alternative mechanisms of action. The AGE-RAGE signaling pathway is a well-recognized mediator of oxidative stress and inflammation (Wu et al., 2025). Activation of this pathway triggers downstream mitogen-activated protein kinase cascades, including MAPK3, leading to excessive reactive oxygen species (ROS) generation (Wu et al., 2025). This, in turn, modulates the expression and activity of apoptosis regulators such as the anti-apoptotic protein BCL2 (Vogler et al., 2025). The consequent mitochondrial outer membrane permeabilization can result in the release of pro-apoptotic factors and the activation of effector proteases like caspases (or their functional homologs, metacaspases, in fungi), ultimately executing programmed cell death (Vogler et al., 2025; Lee et al., 2025). In *A. niger*, functionally homologous components of this pathway are expected to mediate similar stress responses, thus positioning MAPK3 and BCL2 as key nodes for potential antifungal intervention (Garcia et al., 2023; Wu et al., 2025).

Sea buckthorn (*Hippophae rhamnoides* L.) is a resistant shrub that belongs to the family of the Elaeagnaceae (Geetha et al., 2003; Yildiz et al., 2012). Sea buckthorn berries provide seeds that yield SBT seed oil, which contains a large proportion of unsaturated fatty acids, tocopherols, carotenoids, and flavonoids (Basu et al., 2007; Negi et al., 2005; Ting et al., 2011; Ito et al., 2014). It is recognized that SBT oil is safe and has many health benefits for humans (Upadhyay et al., 2009). Previous reviews have highlighted the high safety profile of SBT, classified as a “medicinal and edible homology” substance with low toxicity and low potential for drug resistance in both traditional and modern applications (Dong et al., 2025). Earlier studies have reported the antimicrobial properties of oils that were extracted using different parts of sea buckthorn against food poisoning microorganisms including *Staphylococcus aureus*, *Bacillus subtilis*, *Escherichia coli*, *Bacillus coagulans*, etc. (Yue et al., 2017). Despite the reported antibacterial and antioxidant effects of the SBT extracts, the antifungal mechanism of SBT seed oil has not been studied so far. Our previous research has shown that SBT oil causes significant damage to the mitochondrial structure and function of *A. niger* in a dose-dependent manner, with the most pronounced effects observed at a concentration of 24 mL/L in the culture medium (Xin et al., 2023). This finding suggests its potential as an effective antifungal agent that targets key fungal organelles.

Transcriptomics has become an invaluable tool in the field of antimicrobial studies, as it has been instrumental in the detailed examination of global gene expression changes in line with therapy, thereby determining the potential drug targets and drug mechanisms of action (Domínguez et al., 2017). Natural products SBT oil with its variety of active constituents often have a synergetic effect on various targets and pathways. Network pharmacology offers a powerful approach for dissecting the complex interactions and elucidating the holistic mechanisms of action of natural products (Li et al., 2021). When integrated with transcriptomics, this strategy provides a robust framework for identifying bioactive metabolites and delineating their mechanisms of action in natural products (Hu et al., 2022). The integrated methodology enhanced by molecular docking, MD simulations, and experimental validation is used in this study to comprehensively investigate the molecular mechanism of antifungal activity of SBT seed oil against *A. niger*.

## 2 Materials and methods

### 2.1 Materials

*Aspergillus niger* strain (ACCC 32291), which was used in the present study, was obtained in the Agricultural Culture Collection of China (ACCC). The commercial SBT seed oil was extracted using supercritical CO_2_ fluids at Shanxi Shandi sunshine food Co., Ltd. in Shanxi, China.

### 2.2 Cell treatment and sample preparation

The spore suspension of *A. niger* was prepared following the methodology outlined in the previous study and adjusted to 1 × 10^6^ spores/mL using a hemocytometer (Ben Miri et al., 2023). In the experimental group, SBT seed oil was solubilized in ≤0.1% dimethyl sulfoxide (DMSO), which was diluted in sterile 0.9% physiological saline, resulting in a final concentration of 24 mL/L in the spore suspension. The culture was maintained at 25 °C on a rotary shaker operating at 150 rpm for a duration of 72 h to facilitate optimal interaction between the oil and spores. The control group comprised a spore suspension that received an equivalent volume of vehicle (≤0.1% DMSO), maintained under the same incubation conditions. All subsequent cultures, harvests, and assay preparations utilized aliquots from these identically standardized spore or mycelial suspensions to ensure uniform cell density across all experimental replicates and wells.

### 2.3 Construction of a secondary metabolite library of SBT seed oil and screening of active ingredients

The chemical constituents of SBT seed oil were sourced from databases such as TCMSP and CNKI, utilizing “sea buckthorn seed oil” and “*Hippophae rhamnoides* seed oil” as search terms. The canonical SMILES structures of the identified compounds were sourced from PubChem. Bioactive compounds were evaluated through the SwissADME platform according to two primary criteria: (1) elevated gastrointestinal absorption (GI absorption = “High”), and (2) compliance with a minimum of four out of the five established drug-likeness criteria: Lipinski’s rule, Ghose’s parameters, Veber’s guidelines, Egan’s principles, and Muegge’s standards. Furthermore, compounds exhibiting an oral bioavailability (OB) of 30% or greater, along with a drug-likeness (DL) index of 0.18 or higher as per the TCMSP database, were selected for subsequent analysis.

This study emphasizes the identification of individual bioactive components, while also recognizing the potential for synergistic interactions among the constituents present in SBT seed oil. Future investigations will thoroughly explore these interactions via combination experiments. For example, it will be essential to co-treat *A. niger* with varying ratios of the key fatty acids identified (myristic acid, palmitic acid, palmitoleic acid) at different concentrations and to compare the effects on fungal growth, oxidative stress, and apoptosis against single-component treatments. Moreover, sophisticated network pharmacology models that integrate component-component interactions could be established to offer a more comprehensive insight into the synergistic mechanisms.

### 2.4 Prediction targets and collection and mapping of targets for *Aspergillus niger*

The SMILES notations of the bioactive compounds that were screened were submitted to the SwissTargetPrediction database to predict their potential protein targets, specifically for the species “*Homo sapiens*.” Duplicate targets were eliminated to create a distinct list of potential SBT oil targets. Targets associated with disease for aspergillosis were obtained from four databases: GeneCards, OMIM, TTD, and DrugBank, employing “aspergillosis” as the search term. The targets from these databases were integrated, and duplicates were eliminated to establish a comprehensive list of aspergillosis-related targets. All target protein names were standardized to their official gene symbols utilizing the UniProt database. The overlap between the SBT oil target list and the aspergillosis target list was determined through a Venn diagram, revealing potential targets for SBT oil in the context of aspergillosis. A network focused on compound-target interactions was visualized utilizing Cytoscape software (version 3.9.1).

### 2.5 Protein–protein interaction network construction

The targets outlined in section 2.4 were imported into the STRING database (version 11.5), specifying “*Homo sapiens*” as the organism and establishing a minimum interaction score threshold of 0.700, indicating high confidence. The resultant protein–protein interaction (PPI) network has been successfully acquired. The interaction data was acquired in TSV format and subsequently imported into Cytoscape 3.9.1 for comprehensive analysis. The CytoNCA plugin facilitated the computation of network centrality measures, including the closeness centrality, betweenness centrality, and degree. Targets that ranked in the top 25% by degree were identified as hub targets for further analysis.

### 2.6 GO and KEGG analysis

An enrichment analysis was performed for Gene Ontology (GO), which includes the categories of Biological Process (BP), Molecular Function (MF), and Cellular Component (CC), as well as a Kyoto Encyclopedia of Genes and Genomes (KEGG) pathway enrichment analysis. This was carried out on the shared targets using the clusterProfiler package (version 4.0.5) in R (version 4.1.2). Statistical significance was determined for terms with a corrected *p*-value (Benjamini–Hochberg method) of ≤0.05. The results were illustrated utilizing the ggplot2 package. A target-pathway network was developed using Cytoscape (version 3.9.1) to depict the connections between essential targets and enriched pathways.

### 2.7 Molecular docking

Molecular docking was conducted to assess the interactions between the leading active compounds (as detailed in section 2.3) and the core target proteins (outlined in section 2.5) employing a semi-flexible docking methodology. The three-dimensional structures of the ligands were acquired in Mol2 format from PubChem or optimized with Avogadro (version 1.2.0) and subsequently energy-minimized utilizing the MMFF94 force field. Protein crystal structures for the key targets (e.g., MAPK3/ERK1: PDB ID 4QTB, 1.80 Å; CASP3: PDB ID 5I9B, 1.63 Å; BCL2: PDB ID 6IE8, 1.80 Å) were obtained from the RCSB PDB database. Criteria for selection encompassed a resolution of less than 2.5 Å, human origin or high homology, and the structural integrity of the binding site. Proteins were prepared using AutoDock Tools (version 1.5.7) by removing water molecules, adding polar hydrogens, and assigning Kollman charges. The docking grids were strategically centered on the established active site of each protein, with dimensions carefully designed to encompass the ligands effectively. Docking simulations were conducted utilizing AutoDock Vina (version 1.1.2). For each ligand-protein pair, a total of 10 docking runs were performed, and the pose with the most favorable (lowest) binding energy was selected for subsequent analysis. The binding interactions were visualized using PyMOL (version 2.5.0) and Discovery Studio Visualizer (version 2021).

### 2.8 Molecular dynamic simulation

Molecular dynamics simulations were conducted on the top-ranked protein-ligand complexes identified via docking, employing GROMACS (version 2022.4). The AMBER14SB force field was employed for the protein, while the GAFF2 force field, accompanied by AM1-BCC charges assigned through the ACPYPE server, was utilized for the ligands. Each complex was surrounded by a cubic box of TIP3P water molecules, maintaining a minimum distance of 1.0 nm from the protein surface. Na^+^ or Cl^−^ ions were introduced to achieve system neutralization. Energy minimization was performed using the steepest descent algorithm until the maximum force decreased to below 1,000 kJ/mol/nm. The system was then equilibrated using NVT (constant number of particles, volume, and temperature) and NPT (constant Number of particles, pressure, and temperature) ensembles for a duration of 100 ps each. The temperature was maintained at 298 K utilizing the v-rescale algorithm, while the pressure was regulated to 1 bar through the Berendsen barostat. Production molecular dynamics simulations were conducted for a duration of 100 ns under NPT conditions, utilizing a time step of 2 fs. The particle mesh Ewald (PME) method was employed to address long-range electrostatic interactions, while a cutoff of 1.2 nm was implemented for short-range van der Waals and Coulomb interactions. All bonds that include hydrogen atoms were constrained utilizing the LINCS algorithm, employing an integration step size of 2 fs. Trajectories were recorded at intervals of 10 ps for further examination.

The assessment of the stability of the protein-ligand complexes involved calculating the root mean square deviation (RMSD) for both the protein backbone and the ligand. The calculation of root mean square fluctuation (RMSF) for protein residue alpha-carbons was performed to assess local flexibility. The formation of hydrogen bonds between the ligand and the protein was systematically tracked during the simulation period. The molecular mechanics/generalized Born surface area (MM/GBSA) method was employed to calculate the binding free energy (ΔGbind) for each complex using the gmx_MMPBSA tool, with 100 frames extracted at regular intervals from the stabilized phase of the simulation. The solvent-accessible surface area (SASA) and the radius of gyration (Rg) were determined to evaluate the compactness and stability of the complexes.

### 2.9 Transcriptome sequencing and analysis

Samples were submitted to Shanghai Painuosheng for sequencing. Following RNA extraction, purification, and library construction, the library was quality-checked and underwent double-ended sequencing. Following sequencing, the raw dataset, comprising 3′ end spliced sequence and low-quality entries, was meticulously filtered out. The remaining data were processed to yield effective transcript data for subsequent analysis. The FPKM value of each gene was taken as a reference, based on which the expression level of the transcript was computed. Besides filtering the differentially expressed genes with the characteristic of expression difference of greater than |log2FoldChange| >1 and significance *p*-value <0.05, we will take into consideration the overall expression levels of the genes. Genes with low absolute expression level may be of limited biological relevance even if they pass the above fold-change and *p*-value criteria. Thus, genes with an average FPKM value of more than 10 in any group will be given priority consideration. In the meantime, gene function annotation will be incorporated. Genes associated with known biological processes involved in fungal growth, stress response, and metabolism will be prioritized for screening, with the objective of systematically and precisely identifying critical differentially expressed genes. Enrichment analysis and metabolic pathway analysis were conducted on the GO terms and KEGG pathways corresponding to the DEGs.

### 2.10 Determination of AGEs

To quantify endogenous advanced glycation end-products (AGEs) in *A. niger* cells using a commercial ELISA kit, AGE-BSA standards were prepared in-house as a reference standard for quality control and curve validation: bovine serum albumin (BSA; Sigma, Cat. # A7030) was incubated with 20 mM DL-glyceraldehyde (Sigma, Cat. # G5001) at 37 °C for 1 week in phosphate-buffered saline (PBS; Sigma, Cat. # P3813, pH 7.4). The successful glycation of BSA was confirmed by a significant increase in fluorescence intensity (excitation 370 nm, emission 440 nm) compared with the non-glycated BSA control (incubated identically without DL-glyceraldehyde). The resulting mixture was subjected to five rounds of dialysis against PBS at 4 °C, with each round lasting 2 h, to remove excess reactants. The sample was then concentrated by centrifugation at 3,000 rpm for 30 min at 4 °C employing an Amicon Ultra centrifugal filter unit (Millipore, Cat. #UFC910024) (Ko et al., 2010).

Mycelia harvested from 10 mL cultures (initiated at 1 × 10^6^ spores/mL and grown for 72 h) were centrifuged (4,200 rpm, 30 min), washed twice with PBS, homogenized on ice in PBS (0.2 g wet weight in 1.8 mL), and total protein quantified by BCA assay. Lysates were adjusted to 1 mg/mL protein; 50 μL (50 μg protein equivalent) was loaded per well in the 96-well plate. The concentration of AGEs was determined using a commercial AGEs ELISA kit (abbexa, Cat. # abx054078) according to the manufacturer’s instructions. The absorbance was measured at 450 nm using a microplate reader (Biotek, Cat. Synergy HTX), with cell-free wells serving as blanks.

### 2.11 Determination of the mitochondrial membrane potential

The JC-1 staining working solution was prepared by first mixing 25 μL of the 200× JC-1 Stock Solution (Sigma-Aldrich, Cat. #T4069) with 4 mL of ultrapure water and then adding 1 mL of the JC-1 staining buffer (5×) to obtain 5 mL of JC-1 staining working solution. Subsequently, 500 μL of *A. niger* mycelial cell suspension (adjusted to 1 × 10^6^ spores/mL by hemocytometer counting from the 72-h culture described in section 2.2) was added to 500 μL of the JC-1 staining working solution, achieving a 1:1 dilution (final concentration of 5 × 10^5^ spores/mL in the mixture). The samples were thoroughly mixed, and 200 μL aliquots were transferred to each well of a 96-well plate (in triplicate), resulting in a final uniform cell density of approximately 1 × 10^5^ spores per well across all wells and replicates. The plate was then subjected to time-scanning analysis using the microplate reader (Agilent Biotek, Cat. #Synergy HTX) with an excitation wavelength of 485 nm and two emission wavelengths: 528 nm (for JC-1 monomers) and 590 nm (for JC-1 aggregates).

### 2.12 Determination of oxidative stress response indicators in *Aspergillus niger*

A 10^8^ spores/mL spore suspension of *A. niger* (prepared from the standardized 72-h culture initiated at 1 × 10^6^ spores/mL and thoroughly homogenized to ensure uniform cell density) was centrifuged at 4,200 rpm for 30 min. The precipitate was selected, washed with PBS (Sigma, Cat. #P3813), and resuspended in 500 μL of PBS. The suspension was supplemented with fluorescent probe DCFH-DA (Beyotime, Cat. #S0033S), which is commonly used to detect intracellular ROS, to a final concentration of 10 μmol/L. The suspension was thereafter incubated at 30 °C with 60 r/min rotary agitation for 4 h, with an equal volume of dimethyl sulfoxide (DMSO) used as the solvent control to account for any potential effects of the probe solvent on mycelial fluorescence or viability. The cells were after incubation centrifuged to discard the supernatant of the probe. The mycelia pellet was washed with PBS and suspended in 500 μL of PBS. The fluorescence intensity of the resuspended mycelia was directly measured with a multifunctional microplate reader (Agilent BioTek, Cat. #Synergy HTX) at 485 nm excitation wavelength and at 528 nm emission wavelength.

The level of MDA, an indicator of lipid peroxidation, was determined using a commercial MDA assay kit (Beyotime, Cat. # S0131S) following the manufacturer’s guidelines. This assay relies on the MDA-TBA reaction to form a pink-colored chromophore, and its absorbance was recorded at 535 nm (Zhao et al., 2024).

### 2.13 Enzyme activity assay

Following the 72-h incubation as described in section 2.2, the *A. niger* mycelia were harvested by centrifugation at 4,200 rpm for 30 min at 4 °C. A mycelial sample (0.2 g) was accurately weighed and homogenized on ice using a mechanical homogenizer with 1.8 mL of pre-chilled, sterile 0.9% sodium chloride (NaCl) solution (Procell, Cat# PB180353). The homogenate was centrifuged at 10,000 rpm for 15 min at 4 °C to remove cellular debris, and the resulting supernatant was collected as the crude enzyme extract and kept on ice for immediate analysis. The activities of key mitochondrial enzymes—succinate dehydrogenase (SDH) and ATP synthase (ATPase)—were determined as indicators of mitochondrial metabolic status; SDH is the integral component of the tricarboxylic acid (TCA) cycle and electron transport chain, reflecting respiratory capacity and oxidative metabolism, while ATPase evaluates ATP synthesis capacity, thereby serving as established markers for mitochondrial dysfunction and energy deficits (Shinde et al., 2006). These activities were determined using commercial assay kits according to the manufacturers’ instructions. Specifically, SDH activity was measured using the Succinate Dehydrogenase Activity Assay Kit (Abcam, Cat. # ab228560), and ATPase activity was evaluated using the ATPase Assay Kit (Colorimetric) (Abcam, Cat. # ab234055).

### 2.14 Real-time fluorescence quantitative PCR analysis

Transcript levels of AGE-RAGE signaling pathway genes in *A. niger* after SBT seed oil exposure were measured by one-step RT-qPCR. RNA samples were analyzed using the CellsDirect^™^ One-Step qRT-PCR Kit (Thermo Fisher, Cat. # 11753500) on a QuantStudio^™^ 5 instrument (Thermo Fisher, Cat. # A34322). The purity of the extracted RNA was assessed using a NanoDrop spectrophotometer (Thermo Fisher Scientific), with A260/A280 ratios ranging from 1.8 to 2.0 indicating high purity. Primers for *MAPK3* and *METACASP* were designed in Primer Premier 5.0 (Premier Biosoft). The following primer pairs were used: *MAPK3*-F: 5′-AGGTTTGCTGAAGAAACGGCGAAC-3′, *MAPK3*-R: 5′-CCTTCTTCAACACCTTGACAGCGTAG-3′; *METACASP*-F: 5′-AGGCGGTCACGCTAAATATTAGAGC-3′, *METACASP*-R: 5′-TTTCCATTTCGACGATCCAGTCGTC-3′. All primers passed in silico specificity screening against the *A. niger* genome and secondary structure evaluation via the OligoAnalyzer Tool (Integrated DNA Technologies).

Amplifications were run in 20 μL reactions comprising 10 μL of 2 × Reaction Mix, 1 μL each of forward and reverse primers (10 μM), 2 μL RNA (50 ng/μL) and nuclease-free water to final volume. The thermal protocol included cDNA synthesis (50 °C, 15 min), pre-denaturation (95 °C, 2 min), then 40 cycles of 95 °C for 15 s and 60 °C for 30 s. All samples and no-template controls were run in triplicate. The *A. niger* β-actin gene served as the internal control, and expression fold-changes were derived by the 2^−ΔΔCT^ algorithm using QuantStudio Design & Analysis Software v1.5.1 (Thermo Fisher).

### 2.15 Statistical analysis

All statistical analyses were performed using R software (v4.1.2) or GraphPad Prism (v9.0). Statistical significance was defined as a *p*-value <0.05, unless otherwise specified for multiple testing corrections. The specific methods for each experimental approach are detailed below.

#### 2.15.1 Network pharmacology and transcriptomic enrichment analysis

For GO and KEGG pathway enrichment analyses in both network pharmacology and transcriptomics, *p*-values were adjusted for multiple comparisons using the Benjamini–Hochberg false discovery rate (FDR) procedure. Terms with an adjusted *p*-value (FDR) <0.05 were considered statistically significant.

#### 2.15.2 Transcriptomic differential expression analysis

Differential expression analysis of RNA-seq data was conducted using the “DESeq2” package in R. Genes were identified as DEGs if they met the thresholds of |log₂FoldChange| >1 and an adjusted *p*-value (FDR) <0.05.

#### 2.15.3 Molecular docking and dynamics simulations

The binding affinity (kcal/mol) from molecular docking was reported as the lowest energy pose from 10 independent runs for each ligand-receptor pair. Parameters from MD simulations, including RMSD, Rg, and SASA, are presented as mean ± SD calculated from the simulation trajectories. The binding free energy (ΔGbind) was calculated using the MM/GBSA method on 100 frames extracted from the stable phase of the simulation and is reported as mean ± SD.

#### 2.15.4 *In vitro* experimental data

Data from all *in vitro* experiments (e.g., ELISA, enzymatic activity assays, ROS/MDA quantification, qPCR) were obtained from three independent biological replicates (*n* = 3) and are presented as mean ± SD. The normality of data distribution and homogeneity of variances were assessed using the Shapiro–Wilk test and *F*-test, respectively. For comparisons between two groups (control vs. SBT seed oil treatment), a two-tailed Student’s *t*-test was used for data conforming to parametric assumptions. The non-parametric Mann–Whitney *U* test was applied when these assumptions were violated. The following conventions were used to denote significance levels in figures: ^*^p < 0.05, ^**^p < 0.01, ^***^p < 0.001, and ^****^p < 0.0001.

## 3 Results

### 3.1 Network pharmacology analysis reveals key compounds, targets, and pathways of SBT seed oil against *Aspergillus niger*

**Figure 1:**
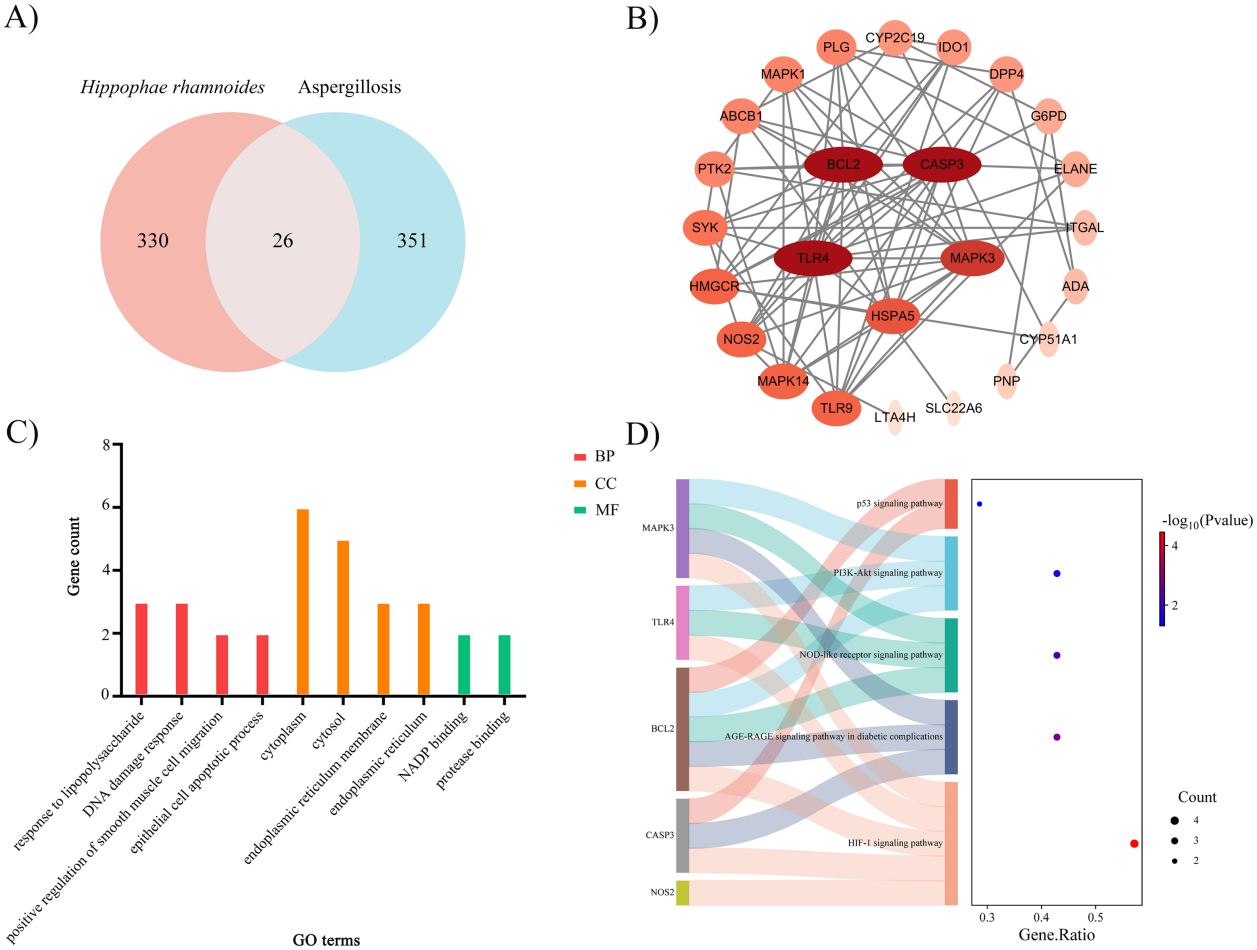
Investigation of the anti-*Aspergillus niger* mechanism of SBT seed oil via network pharmacology. **(A)** Identification of shared targets between SBT seed oil and *A. niger* using a Venn diagram. **(B)** Topological analysis of the protein–protein interaction (PPI) network. Nodes represent target proteins, colored by their betweenness centrality to highlight potential key targets. Edges represent protein interactions. **(C)** Gene Ontology (GO) term enrichment analysis for the shared targets, displaying significantly enriched biological functions (adjusted *p* < 0.05, Benjamini–Hochberg correction). **(D)** Kyoto Encyclopedia of Genes and Genomes (KEGG) pathway enrichment analysis, visualizing significantly enriched pathways (adjusted *p* < 0.05, Benjamini–Hochberg), where dot size represents gene count and color represents adjusted *p*-value. Analyses were performed using Cytoscape (v3.9.1), STRING database (v11.5), and clusterProfiler package (v4.0.5) in R (v4.1.2).

Fifteen active compounds of SBT seed oil were identified for network pharmacology analysis (Table 1). After the screening of the potential targets for the 15 compounds and removal of duplicates or compounds with unknown targets, a novel list with 7 active compounds and 356 putative targets was established for further analysis. Moreover, 377 disease targets of A. Targets of Niger were identified, and a combined list of 26 targets associated with SBT seed oil was achieved (Figure 1A). The network plot gave a total of 55 interactions among the active compounds and 26 targets. Based on this network analysis, the four highest degree values compounds were identified as follows: myristic acid (S1), which targets 10; palmitic acid (S2), which targets 4; palmitoleic acid (S3), which targets 3; and triglycerides (S8), which targets 2.

**Table 1:** Information on the effective compounds of SBT seed oil.

No.	Name	Literature reference
S1	Myristic acid	Seed oil quantity and fatty acid composition of different sea buckthorn (*Hippophae rhamnoides* L.) wild populations in Iran
S2	Palmitic acid	Seed oil quantity and fatty acid composition of different sea buckthorn (*Hippophae rhamnoides* L.) wild populations in Iran
S3	Palmitoleic acid	Seed oil quantity and fatty acid composition of different sea buckthorn (*Hippophae rhamnoides* L.) wild populations in Iran
S4	Stearic acid	Seed oil quantity and fatty acid composition of different sea buckthorn (*Hippophae rhamnoides* L.) wild populations in Iran
S5	Oleic acid	Seed oil quantity and fatty acid composition of different sea buckthorn (*Hippophae rhamnoides* L.) wild populations in Iran
S6	Linoleic acid	Seed oil quantity and fatty acid composition of different sea buckthorn (*Hippophae rhamnoides* L.) wild populations in Iran
S7	Linolenic acid	Seed oil quantity and fatty acid composition of different sea buckthorn (*Hippophae rhamnoides* L.) wild populations in Iran
S8	Triglyceride	Structure of chemical compounds, methods of analysis and process control
S9	Tocopherol	The physicochemical composition of sea buckthorn (*Hippophae rhamnoides* L.) oil and its treatment characteristics
S10	Β-Tocotrienol	The physicochemical composition of sea buckthorn (*Hippophae rhamnoides* L.) oil and its treatment characteristics
S11	Phytosterol	The physicochemical composition of sea buckthorn (*Hippophae rhamnoides* L.) oil and its treatment characteristics
S12	Diglyceride	Fatty acid composition of lipids in sea buckthorn (*Hippophae rhamnoides* L.) berries of different origins
S13	Ethyl dodecacrylenate	Essential oil and fatty acid composition of the fruits of *Hippophae rhamnoides* L. (sea buckthorn) and *Myrtus communis* L. from Turkey
S14	Ethyl caprylate	Essential oil and fatty acid composition of the fruits of *Hippophae rhamnoides* L. (sea buckthorn) and *Myrtus communis* L. from Turkey
S15	Decyl alcohol	Essential oil and fatty acid composition of the fruits of *Hippophae rhamnoides* L. (sea buckthorn) and *Myrtus communis* L. from Turkey

These compounds may be considered the predominant active constituents of SBT seed oil against *A. niger*. The database of SPRING was used to construct a protein–protein interaction (PPI) network to identify the candidate targets. The network consisted of 26 nodes and 80 edges. MAPK3, HMGCR, CASP3, BCL2, HSPA5, NOS2, and TLR4 possessed the maximum degree values during the target analysis and were identified as potentially crucial targets (Figure 1B).

GO enrichment analysis identified that these 7 high-ranking targets were significantly enriched in 12 biological processes, 4 cellular component terms, and 2 molecular functions (*p* < 0.05). The most enriched terms for biological processes were response to lipopolysaccharides, DNA damage response, regulation of smooth muscle cell migration by positive regulation, and apoptosis of epithelial cells, among others. At the cellular component level, cytoplasm, endoplasmic reticulum membrane, endoplasmic reticulum, and cytoplasm were the most enriched terms. The most enriched molecular function terms were NADP binding and protease binding (Figure 1C). Thirty-two KEGG pathways were associated with these 7 central target proteins, 14 of which were significantly enriched (*p* < 0.05). The identified pathways are the HIF-1 signaling pathway, AGE-RAGE-like signaling pathway in *A. niger,* NOD-like receptor signaling pathway, PI3K Akt signaling pathway, and p53 signaling pathway (Figure 1D). Together, network pharmacology analysis presents a valuable pathway for future research into the underlying mechanism.

### 3.2 Transcriptome analysis reveals SBT seed oil induces extensive gene expression changes in redox processes and metabolic pathways

**Figure 2:**
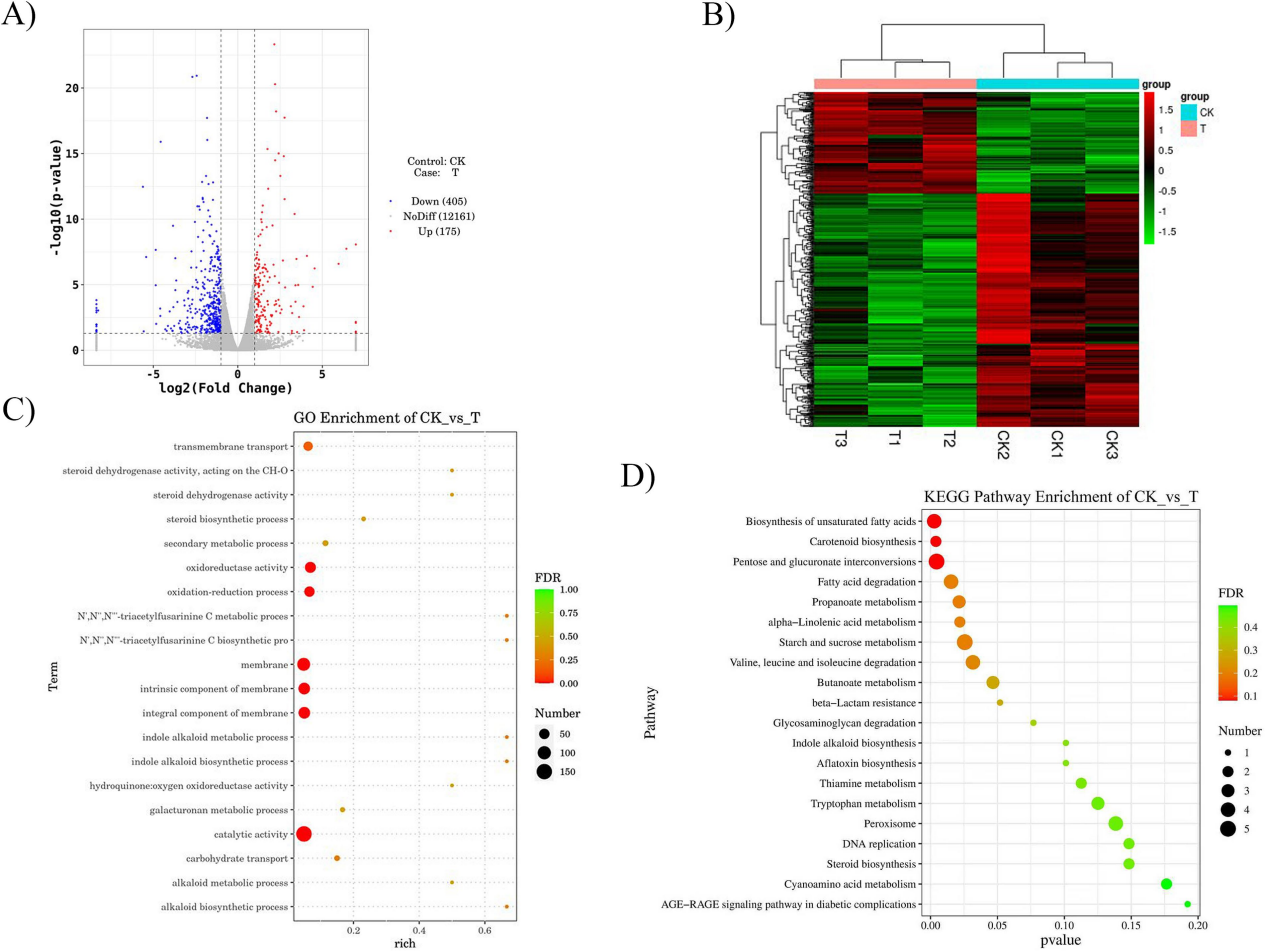
Transcriptome sequencing analysis of the antifungal mechanism of SBT seed oil against *A. niger*. **(A)** Volcano plot visualizing the differentially expressed genes (DEGs) between the SBT seed oil-treated and control groups. **(B)** Hierarchical clustering heatmap of DEGs, showing distinct transcriptional profiles between conditions. **(C)** Gene Ontology (GO) enrichment analysis highlighting the significantly overrepresented biological functions among the DEGs (adjusted *p* < 0.05, Benjamini–Hochberg). **(D)** KEGG pathway enrichment analysis identifying the key signaling pathways affected by SBT seed oil (adjusted *p* < 0.05, Benjamini–Hochberg). Sequencing was performed on three independent biological replicates per group from the 72-h culture (control vs. 24 mL/L SBT seed oil in ≤0.1% DMSO vehicle). DEGs were identified using DESeq2 in R with |log₂FoldChange| >1 and adjusted *p* < 0.05.

Figures 2A,B illustrates the DEGs between groups. In contrast to the control group, the experimental group possessed a total of 580 DEGs, which consisted of 175 upregulated and 405 downregulated genes. GO enrichment analysis was conducted on the DEGs and categorized into three categories: molecular function, biological process, and cellular component. The genes that were identified to have differential expression based on molecular functions were predominantly related to catalytic activity regulation, oxidoreductase activity, steroid dehydrogenase activity, and hydroquinone oxygen oxidoreductase activity. Redox processes, transmembrane transport, and carbohydrate transport were significantly enriched after the SBT treatment (Figure 2C). KEGG analysis indicated that out of the 54 pathways analyzed, differentially expressed genes (DEGs) were predominantly associated with physiological metabolism and connected pathways. These were the directions indicating enrichment following SBT seed oil supplementation and were primarily biosynthesis of unsaturated fatty acids, carotenoid biosynthesis, AGE-RAGE-like signaling pathway in *A. niger*, pentose and glucuronic acid metabolic conversion, fatty acid degradation pathway, starch and sucrose metabolism pathway, and the amino acid degradation pathway, among others (Figure 2D).

### 3.3 Transcriptomic mapping reveals SBT seed oil reprograms central carbon, lipid, and amino acid metabolism in *Aspergillus niger*

**Figure 3:**
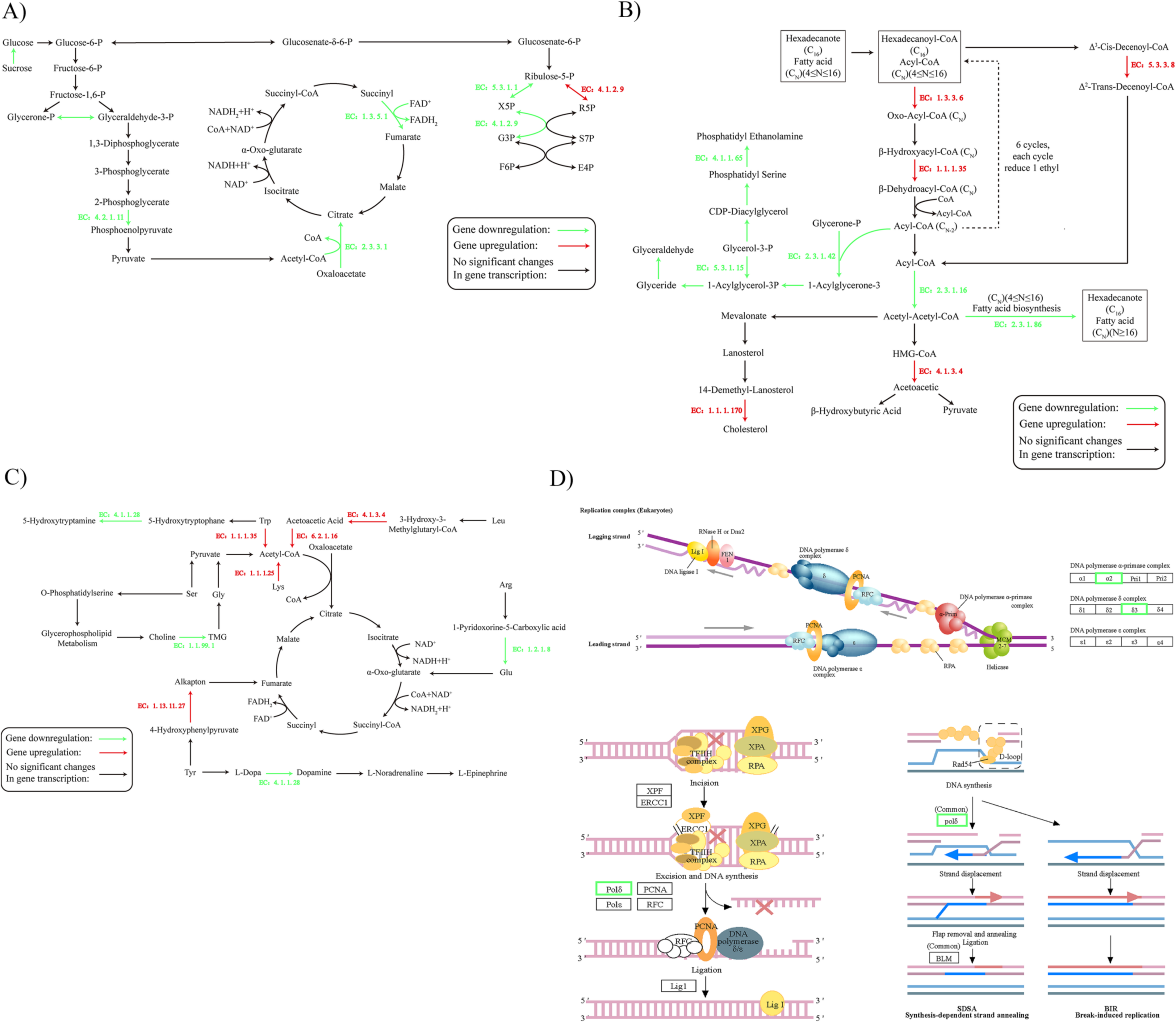
Integrative view of metabolic dysregulation in *A. niger* induced by SBT seed oil through transcriptome network analysis. **(A)** Network of central carbon metabolic pathways. **(B)** Network of lipid metabolic pathways. **(C)** Network of amino acid and protein metabolic pathways. **(D)** Network of nucleotide and genetic information metabolic pathways. Pathways were constructed based on significantly differentially expressed genes (|log₂FoldChange| >1, adjusted *p* < 0.05) from the same RNA-seq dataset as Figure 2 (control vs. 24 mL/L SBT seed oil in ≤0.1% DMSO vehicle).

To delineate the impact of SBT seed oil on the central metabolism of *A. niger*, we constructed a comprehensive map of transcriptomic changes across core metabolic pathways (Figure 3). The analysis revealed a widespread disruption in energy metabolism and biosynthetic processes.

Energy metabolism is severely compromised. Treatment with SBT seed oil led to a concerted downregulation of genes involved in carbohydrate transport and glycolysis, including enolase (EC:4.2.1.11), indicating impaired glucose utilization (Figure 3A). This was coupled with the suppression of key tricarboxylic acid (TCA) cycle enzymes, citrate synthase (EC:2.3.3.1) and succinate dehydrogenase (EC:1.3.5.1), pointing to a severe deficit in energy production. The concurrent downregulation of ATPase activity further confirms this energy crisis.

Lipid metabolism is redirected from synthesis to catabolism. Facing energy depletion, *A. niger* appears to shift lipid metabolism toward degradation for energy salvage. Genes encoding β-oxidation enzymes, such as alkanoyl CoA oxidase (EC:1.3.3.6), were upregulated (Figure 3B). Conversely, genes involved in the synthesis of fatty acids (e.g., fatty acid synthase, EC:2.3.1.86) and structural phospholipids (e.g., phosphoglycerol-O-acyltransferase, EC:2.3.1.42) were significantly downregulated, suggesting disruption of cell membrane integrity and biogenesis.

Amino acid metabolism is rewired to replenish central metabolism. The metabolism of several amino acids, including leucine and tyrosine, was altered, leading to an increased production of acetyl-CoA and TCA cycle intermediates like fumarate (Figure 3C). This suggests a compensatory mechanism to replenish the crippled central carbon metabolism. Additionally, the downregulation of aromatic amino acid decarboxylase (EC:4.1.1.28) indicates a potential shutdown of secondary metabolic branches to conserve resources.

Genetic information processing and cell proliferation are inhibited. The metabolic stress culminated in a suppression of cell proliferation. We observed downregulated expression of genes critical for DNA replication (e.g., DNA polymerase subunits) and repair mechanisms (Figure 3D). Furthermore, the expression of ribosomal protein genes and key enzymes in mRNA synthesis was suppressed, indicating a global reduction in transcriptional and translational capacity.

In summary, SBT seed oil triggers a global metabolic crisis in *A. niger*, characterized by blocked energy production, a catabolic shift in lipid metabolism, and a consequent arrest of growth and proliferation.

### 3.4 Integration of network pharmacology and transcriptomics identifies the AGE-RAGE pathway and validates core compound-target interactions via molecular docking

**Figure 4:**
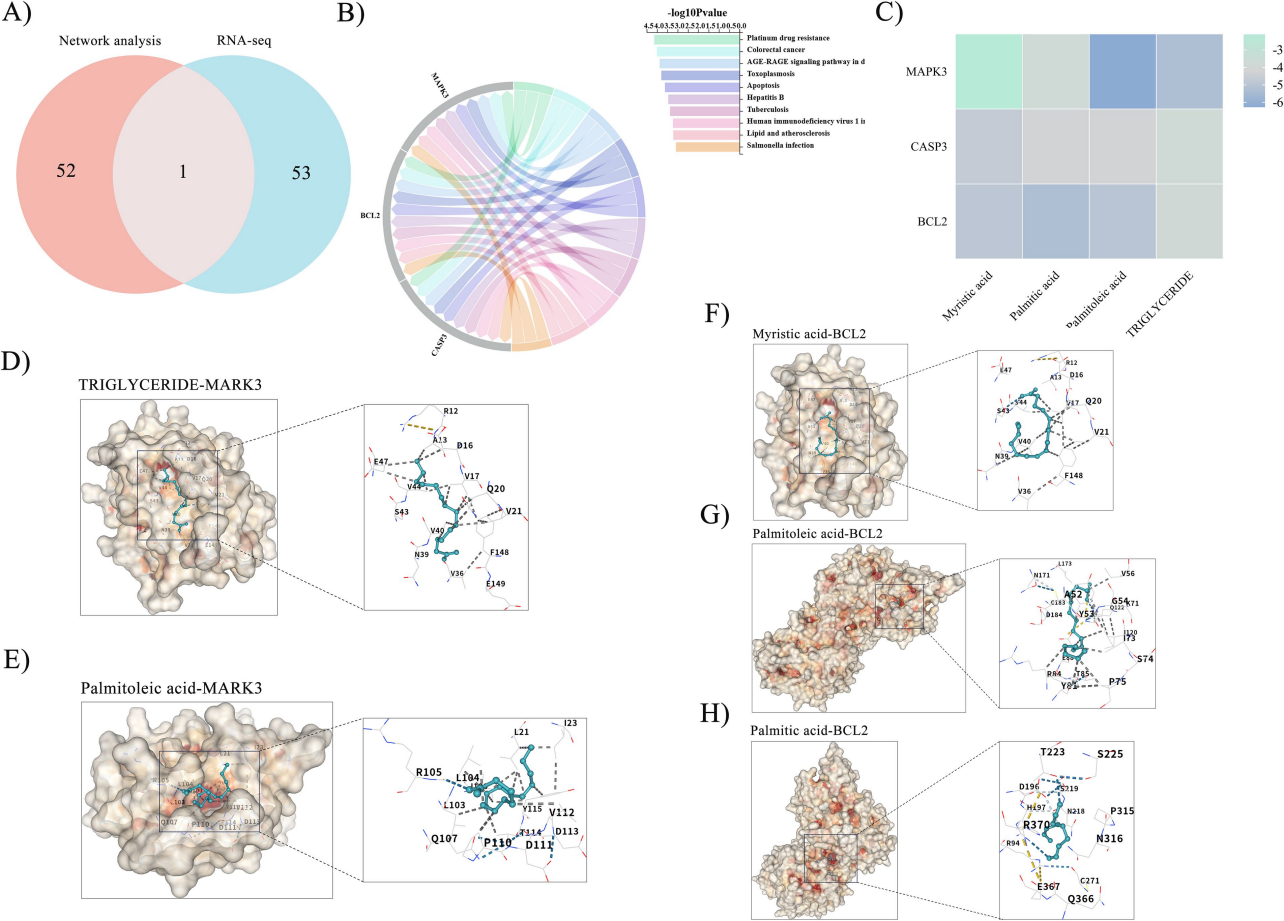
Integration of network pharmacology, transcriptomics, and molecular docking validation for SBT seed oil. **(A)** Intersection of signaling pathways identified by network pharmacology and RNA-seq. **(B)** Chord diagram depicting the enrichment of pathways among the intersection targets. **(C)** Heatmap presenting the binding affinities (kcal/mol) from molecular docking between SBT seed oil components and core targets. Values represent the lowest binding energy from 10 independent docking runs. **(D–H)** Molecular docking poses visualizing the predicted binding modes of specific compounds with their respective protein targets. Docking was performed with AutoDock Vina (v1.1.2) on PDB structures as described in section 2.7.

We further integrated the previously discussed network pharmacology with transcriptomics, and the findings suggest that the AGE-RAGE-like signaling pathway in *A. niger* may represent a promising therapeutic target pathway (Figure 4A). In-depth examination of this intersection signaling pathway indicated that these genes were primarily linked to MAPK3, METACASP, and BCL2 targets, correlating with the network pharmacology and transcriptomic findings outlined previously (Figure 4B). As a result, active compounds associated with the anticipated target proteins were chosen for subsequent examination. These encompass myristic acid, palmitic acid, palmitoleic acid, and triglycerides. The results of the docking binding energies are illustrated in Figure 4C, represented as a heatmap. Palmitoleic acid establishes hydrogen bonds with amino acid residues including (ARG) 84, (GLU) 88, (ILE) 73, (TYR) 53, (LYS) 71, (VAL) 56, (ALA) 69, (LEU) 173, (UNL1) 1, and (ASP) 123 of the MAPK3 target protein. Triglycerides establish hydrogen bonds with amino acid residues, including (UNL1) 1, within the MAPK3 target protein. Myristic acid establishes hydrogen bonds with amino acid residues including (UNL1) 1, (HIS) 121, (GLU) 123, (TYR) 204, (SER) 205, (GLU) 163, (THR) 62, (GLY) 122, (ILE) 126, (CYS) 163, and (ARG) 207 within the BCL2 target protein. Similarly, palmitoleic acid interacts through hydrogen bonds with residues such as (GLU) 76, (LYS) 224, (ASN) 80, (ALA) 227, (LEU) 81, (ASP) 228, (PHE) 275, (TYR) 276, (HIS) 277, and (LYS) 82 of the BCL2 target protein. Palmitic acid establishes hydrogen bonds with amino acid residues including (LEU) 104, (LEU) 21, (ILE) 100, (TYR) 115, (THR) 114, (LEU) 103, and (PRO) 110 within the BCL2 target protein (Figures 4D–H).

### 3.5 Molecular dynamics simulations demonstrate stable binding of SBT compounds to BCL2 and MAPK3, validated by RMSD, Rg, SASA, and binding free energy

**Figure 5:**
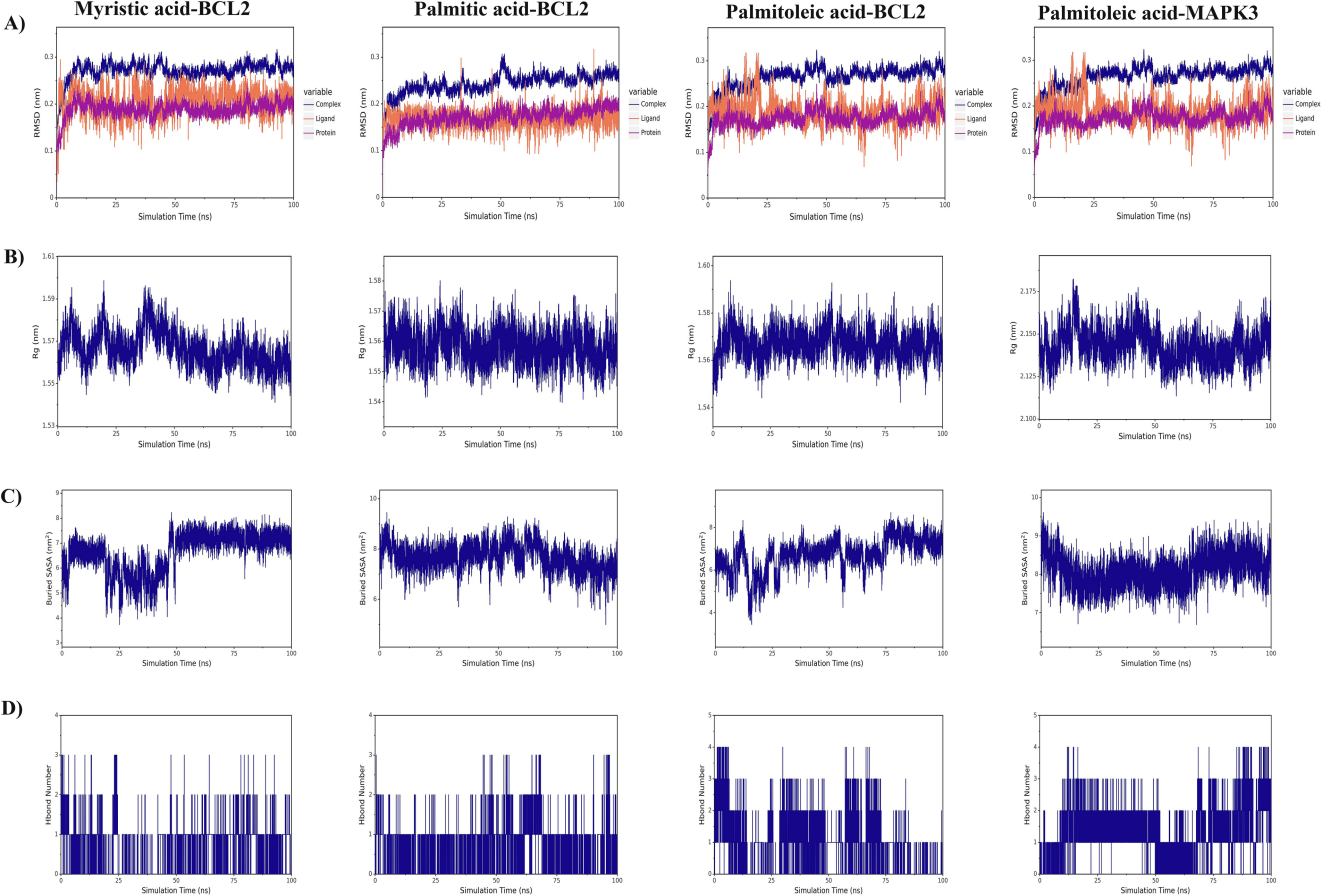
Molecular dynamics simulations of SBT seed oil key compounds and target protein complexes. **(A)** Root mean square deviation (RMSD) values of the four complexes over simulation time. **(B)** Radius of gyration (Rg) values. **(C)** Solvent-accessible surface area (SASA) values. **(D)** Number of hydrogen bonds formed. Simulations were run for 100 ns using GROMACS (v2022.4) with AMBER14SB/GAFF2 force fields. Data are presented as mean ± SD from the stable phase of the 100 ns trajectory.

MD simulations were performed to investigate the stability of protein–ligand interactions involving four specific protein–ligand complexes: myristic acid–BCL2, palmitic acid–BCL2, palmitoleic acid–BCL2, and palmitoleic acid–MAPK3. RMSD values were used to determine the stability of the simulated interactions such that a value of <0.3 nm represents relatively stable protein–ligand interactions under physiological conditions. The RMSD values for the four complexes soon stabilized to values of 0.27 ± 0.0002 nm, 0.24 ± 0.0002 nm, 0.26 ± 0.0002 nm, and 0.19 ± 0.0001 nm (Figure 5A). The compactness of receptor–ligand binding was observed using the radius of gyration (Rg), with stabilized values of 1.35 ± 0.0001 nm, 1.34 ± 0.0001 nm, 1.35 ± 0.0001 nm, and 1.84 ± 0.0001 nm, respectively (Figure 5B). Solvent-accessible surface area (SASA) measurement is a critical parameter for protein folding and stability. SASA values of the four complexes were constant at 94.51 ± 0.02 nm^2^, 92.44 ± 0.02 nm^2^, 93.98 ± 0.02 nm^2^, and 168.05 ± 0.02 nm^2^, respectively (Figure 5C). The percentage of hydrogen bond formation is a critical value for assessing the stability of protein–ligand interaction. Interestingly, the interaction between palmitoleic acid and MAPK3 had the greatest density and intensity of hydrogen bond formation (Figure 5D).

The binding free energies (∆Gbind) values of the four protein–ligand complexes were subsequently evaluated using the MM/GBSA approach, where a lower ∆Gbind value denotes a higher receptor–ligand affinity. The top four complexes with ranking according to the aforementioned values are myristic acid-BCL2 (−98.722 ± 5.522 kJ/mol), palmitic acid-BCL2 (−78.896 ± 2.883 kJ/mol), palmitoleic acid-BCL2 (−99.134 ± 10.929 kJ/mol), and palmitoleic acid-MAPK3 (−49.261 ± 3.934 kJ/mol). Results of molecular docking analysis validate findings reported herein.

### 3.6 SBT seed oil activates the AGE-RAGE-like signaling pathway, inducing mitochondrial dysfunction followed by oxidative stress and apoptosis in *Aspergillus niger*

The apoptotic indicators assessed in this study include loss of mitochondrial membrane potential (ΔΨm), mitochondrial respiratory chain dysfunction (evidenced by reduced SDH activity) and impaired energy metabolism (reflected by decreased ATPase activity). These mitochondrial impairments subsequently drive the overproduction of ROS and elevated MDA levels as a marker of lipid peroxidation, ultimately leading to the upregulated expression of METACASP (the fungal homolog of mammalian caspases).

**Figure 6:**
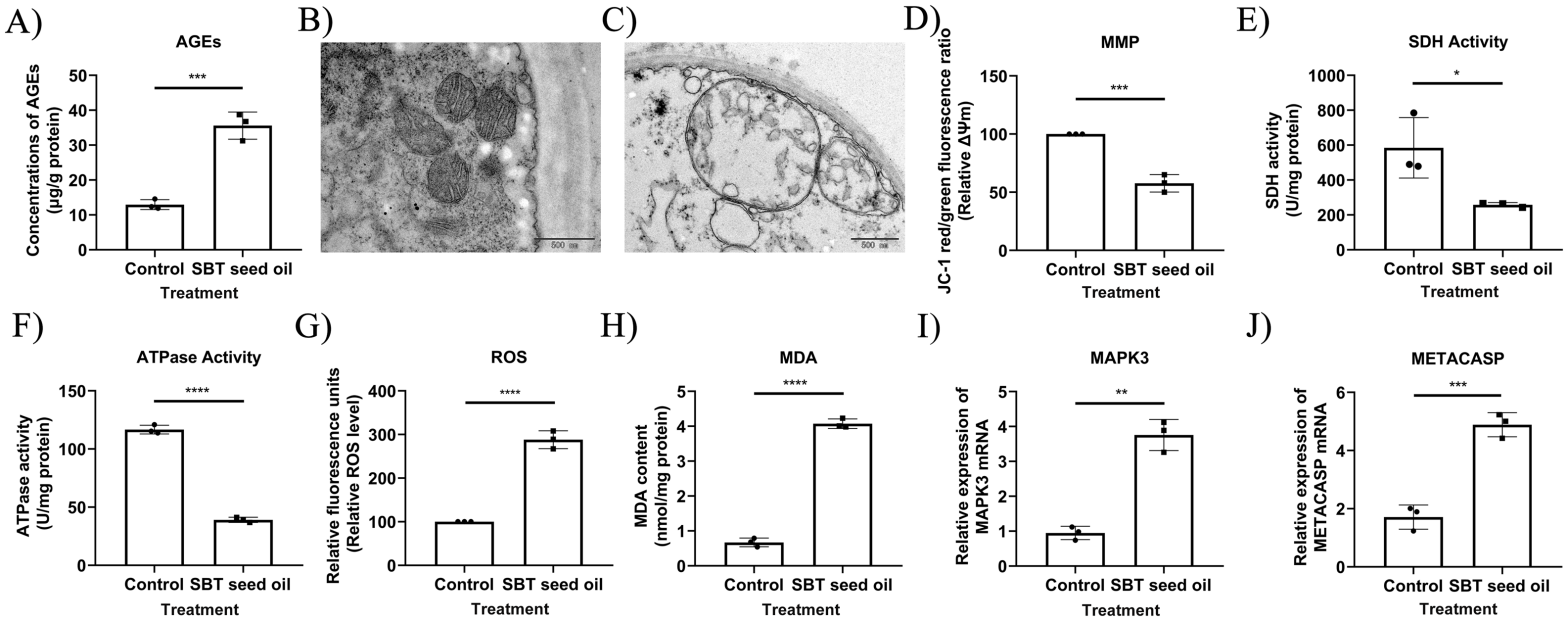
SBT seed oil induces mitochondrial dysfunction and activates the AGE-RAGE-like signaling pathway. **(A)** Concentration of endogenous advanced glycation end products (AGEs) in the control and 24 mL/L SBT seed oil-treated groups. **(B)** Representative transmission electron microscopy (TEM) image (10,000×) showing intact mitochondrial ultrastructure in the control group. **(C)** Representative TEM image (10,000×) showing disrupted mitochondrial ultrastructure (outer membrane rupture, cristae fragmentation, matrix vacuolation) in the SBT seed oil-treated group. **(D)** Measurement of mitochondrial membrane potential (ΔΨm) by JC-1 assay. **(E–H)** Succinate dehydrogenase (SDH) activity, adenosine triphosphatase (ATPase) activity, reactive oxygen species (ROS; DCFH-DA), and malondialdehyde (MDA) levels, respectively, in control vs. treated groups. **(I,J)** Relative mRNA expression levels of MAPK3 and METACASP genes by qRT-PCR. The control group received an equivalent volume of vehicle (≤0.1% DMSO in 0.9% physiological saline). Data are presented as mean ± SD from three independent biological replicates (*n* = 3). Statistical significance was assessed by two-tailed Student’s *t*-test (parametric) or Mann–Whitney *U* test (non-parametric) as described in methods. ^*^p < 0.05, ^**^p < 0.01, ^***^p < 0.001, and ^****^p < 0.0001 vs. control group.

To further elucidate the underlying mechanisms, *A. niger* was used to investigate the effect of SBT seed oil (24 mL/L) and the modulating mechanisms of AGE-RAGE pathway. Endogenous AGE accumulation was first quantified by ELISA (Figure 6A), confirming upstream activation of the AGE-RAGE-like pathway. This led to mitochondrial dysfunction, evidenced by transmission electron microscopy showing outer-membrane rupture, cristae fragmentation and matrix vacuolation (Figures 6B,C), decreased mitochondrial membrane potential (ΔΨm) by JC-1 assay (Figure 6D), and inhibited SDH and ATPase activities (Figures 6E,F). Mitochondrial impairment subsequently drove oxidative stress, manifested as elevated intracellular ROS (DCFH-DA) and lipid peroxidation marker MDA (Figures 6G,H). These changes culminated in apoptotic execution, demonstrated by upregulated mRNA expression of the pathway nodes MAPK3 and the fungal metacaspase homolog METACASP (Figures 6I,J). All assays were performed on the same 72-h treated mycelial samples, supporting the sequential cascade within the AGE-RAGE-like signaling axis.

## 4 Discussion

Fungal infection is an important determinant of human health (Drewniak et al., 2013). While advances have occurred in the treatment of fungal infection, at least from the vantage point of Western medical practices, it is important to note the numerous side effects of antifungal agents (Xu et al., 2013; Sethi et al., 2024). There is thus a need to develop novel therapies for preventing or treating fungal infections. Sea buckthorn seed extracts are known for their strong antibacterial activity owing to their high active ingredient content (Sławińska and Olas, 2022). We initiated an evaluation of SBT seed oil efficacy in the treatment of *A. niger*. We also combined network pharmacology and transcriptomic analysis in the investigation of the mechanism with subsequent validation using molecular docking and experimental techniques.

A thorough network pharmacology investigation was carried out, resulting in the prediction of 26 targets. The results of the GO pathway enrichment analysis show that SBT seed oil suppresses *A. niger* in several biological activities such as lipopolysaccharides, DNA damage response, and epithelial cell apoptosis process. The KEGG pathway analysis proved that the targets were mainly enriched in oxidative stress, the AGE-RAGE signaling pathway, the HIF-1 signaling pathway, and the PI3K-Akt signaling pathway. All these pathways are linked with apoptosis. Oxidative stress is a primary factor contributing to apoptosis (da Silva Dantas et al., 2015). This is characterized by a loss of antioxidation agents such as SOD and a massive response of high-energy oxidative intermediates such as ROS, which escalates apoptosis (Giacco and Brownlee, 2010). Network pharmacology implies that SBT seed oil can hold *A. niger* by increasing oxidative stress and encouraging the process of cellular apoptosis. The main targets that were identified during this process include MAPK3, TLR4, METACASP, BCL2 and HSPA5. Comparable studies with other studies done on the antifungal mechanisms of natural products are informative. The investigation of the antifungal action of tea tree oil against *Botrytis cinerea* and its effects showed that the main interaction is the disruption of mitochondrial functions as an effect caused by the effect on the electron transport chain (Li et al., 2017). Conversely, SBT seed oil seems to interact with many cellular structures and processes that include mitochondria, cell membranes and metabolisms. The differences highlight the unique antifungal effect of SBT seed oil. Our experiment combines network pharmacology, transcriptomics, molecular dynamics with experimental validation and provides a more comprehensive and detailed insight into the antifungal process compared to the earlier works that utilized only one of the methods (Li et al., 2017). Such a multi-omics strategy allows further and more accurate identification of the possible targets and pathways, and it is a significant step forward in natural product-based antifungal studies.

Transcriptome sequencing was subsequently employed to perform more detailed gene-level analysis. The correlation of metabolic pathways revealed that the entire energy metabolism network of *A. niger* significantly changed under SBT seed oil treatment. The total rate of intracellular macromolecular synthesis was decreased. To adapt and ensure a sufficient cellular energy supply, fatty acid synthesis, secondary metabolite synthesis, ribosome synthesis, and cellular transcription are significantly downregulated in cells, whereas fatty acid catabolism is significantly upregulated. Abnormalities in these endogenous pathways can trigger intracellular stress responses, ultimately leading to cell apoptosis (Stoneley et al., 2000; El-Assaad et al., 2010; He et al., 2023). These changes in metabolic network regulation are closely related to the formation of cell membranes and cell walls in *A. niger* cells, and it can be seen that when *A. niger* is stressed by SBT seed oil, the key genes of metabolism (unsaturated fatty acid metabolism, glycerol metabolism, and phospholipid metabolism) related to the synthesis of the cell membrane and the genes of the functional proteins hydrophobic protein, which are closely related to the cell walls, are in the down-regulated state. These phenomena all reveal that cells begin to undergo apoptosis. Furthermore, we pinpointed and examined the biological processes and pathways linked to the genes modulated by SBT seed oil. The AGE-RAGE-like signaling pathway in *A. niger* was significantly enriched in the analysis, aligning with the aforementioned network pharmacological findings. Transcriptomics validated the network pharmacology’s prediction that SBT seed oil promotes cell apoptosis in *A. niger*. Additionally, it demonstrated that SBT seed oil modulated oxidative stress and the expression of specific pathway proteins. Furthermore, we integrated the results of network pharmacology and transcriptome analysis, identifying MAPK3, METACASP and BCL2 as common targets. It is noteworthy that while the network pharmacology prediction, based on human protein databases, identified the apoptosis executor CASP3, the subsequent experimental validation in *A. niger* focused on its fungal functional homolog, METACASP, which is the key effector protein in fungal apoptotic-like cell death. Subsequent enrichment pathway mapping of these cross-targets revealed that the AGE-RAGE-like signaling pathway in *A. niger* was in alignment with the aforementioned findings. BCL2 is an apoptosis inhibitor that can reduce ROS production through various pathways (Deng, 2003). The alteration of BCL2 expression can affect the activity of the ROS signaling pathway. CASP3 is a key executive protein in the process of cell apoptosis, and its expression may affect the sensitivity of cells to oxidative stress (Lin et al., 2016; Zhan et al., 2024). The AGE-RAGE signaling pathway, a classic pathway linked to diabetes complications, was illustrated in all the results mentioned above. MAPK3, CASP3, and BCL2 are all directly implicated in the AGE-RAGE signaling pathway. Therefore, we hypothesized that SBT seed oil exacerbates damage in *A. niger* by modulating the AGE-RAGE-like signaling pathway. Furthermore, we employed molecular dynamics to confirm the interaction between the key compounds and the target proteins identified previously. Based on these findings, we propose that palmitoleic acid could be significant active compounds, whereas BCL2, MAPK3 might serve as crucial binding targets. BCL2 and MAPK3, as effector proteins of the AGE-RAGE signaling pathway, may directly serve as a therapeutic target for SBT seed oil.

Previous studies have demonstrated that the over-activation of the AGE-RAGE signaling pathway induced by AGEs is a pivotal pathway (Yu et al., 2018). AGEs play a crucial role in causing increase oxidative stress (Moldogazieva et al., 2019). Research has shown that the large amount of ROS generated by the activation of the AGE-RAGE signaling pathway directly attacks lipids, proteins, and mitochondrial DNA on the mitochondrial membrane, leading to a decrease in mitochondrial membrane potential and respiratory chain complex activity, thereby affecting the energy metabolism function of mitochondria. ROS can oxidize iron sulfur clusters in mitochondrial respiratory chain complexes, causing their activity to be lost and hindering electron transfer and ATP synthesis (Cortizo et al., 2003; Mahajan and Dhawan, 2013; Albers and Beal, 2000). The signaling pathway activated by RAGE may also interfere with the fusion and division processes of mitochondria, disrupting their normal morphology and function. Abnormal mitochondrial dynamics can lead to mitochondrial fragmentation, affecting its distribution and function within cells, further exacerbating energy metabolism disorders and ROS production (Mao et al., 2018; Elhage et al., 2023). AGE-RAGE can also influence the shift of the mitochondrial Bcl-2/Bax ratio, thereby activating the apoptosis pathway and leading to cell death (Wang et al., 2015). Therefore, we hypothesized that SBT seed oil induces the activation of AGEs in the AGE-RAGE-like signaling pathway in *A. niger* through the relief of the oxidative stress response. This, in turn, modulates BCL2 gene expression and cell apoptosis, and ultimately aggravates myocardial death. Therefore, based on the above findings, we conducted additional experimental confirmation to illuminate the inhibition mechanism of SBT seed oil on *A. niger*. SBT seed oil significantly increased endogenous AGEs, ROS, and MDA levels, and promoted mitochondrial dysfunction and apoptosis-like cell death in *A. niger*. Besides, SBT seed oil significantly regulates the expression of METACASP and MAPK3 of the AGE-RAGE-like signaling pathway in *A. niger*. The observed apoptotic features, including ΔΨm loss, ROS overproduction, MDA accumulation, and METACASP upregulation, collectively support mitochondrial-mediated apoptosis, while SDH inhibition reflects contributing mitochondrial dysfunction. These findings further confirm that SBT seed oil has the ability to enhance oxidative stress and apoptotic damage via the AGE-RAGE-like signaling pathway in *A. niger*. Regarding the safety profile of SBT seed oil, while our study focused on its antifungal mechanisms in *A. niger* without direct cytotoxicity assessments, extensive literature supports its low toxicity. SBT has been recognized as a safe botanical with medicinal and edible homology, demonstrating stable pharmacological effects, high safety, and minimal adverse reactions in animal models and human studies (Dong et al., 2025). For instance, *in vivo* evaluations in rats have shown no significant toxicity at therapeutic doses, with benefits in wound healing and antioxidant activity (Upadhyay et al., 2009). These findings suggest that SBT oil’s mitochondrial-targeted effects in fungi may not translate to mammalian cells, warranting further translational studies but aligning with its established safety for potential clinical applications.

Despite the compelling evidence, there are several limitations in this study. First, the putative targets and pathways were largely predicted by database analyses and computational models, which carry the inherent danger of false positives. Although we validated key findings by experiments, more targeted approaches, e.g., gene knockout or site-directed mutagenesis, are required to ultimately determine the causal roles of individual targets such as MAPK3 and BCL2. Secondly, all our validations were conducted *in vitro*; antifungal activity and the suggested mechanism of SBT seed oil must be further confirmed in *in vivo* models to take into consideration the complexity of a living organism. Future studies should thus be directed at using genetic tools for the precise manipulation of the targets identified in *A. niger* and at establishing animal models of fungal infection to evaluate the therapeutic potential of SBT seed oil in vivo. In addition, separating the individual active compounds, especially palmitoleic acid, and testing their synergistic effects will be important for drug development. Although cytochrome c release was not directly measured, the combined evidence from ΔΨm collapse, ROS accumulation, and METACASP modulation provides robust support for apoptosis. Future studies could include such specific assays to distinguish apoptosis from necrosis more definitively.

## 5 Conclusion

From the basis of an integrated multi-omics approach, this study provides strong evidence that SBT seed oil exerts its antifungal effects against *A. niger* primarily by the AGE-RAGE like pathway (Supplementary Figure 1). Critical constituents—myristic, palmitic, and palmitoleic acids—were *in silico* predicted and dynamically confirmed to stably interact with core targets like MAPK3, METACASP, and BCL2. Transcriptomics demonstrated that treatment causes the overall metabolic reprogramming with disrupted energy metabolism, triggered oxidative stress, and mitochondrial dysfunction that finally triggers apoptosis. Experimental assays confirmed the role of SBT oil in enhancing ROS, lipid peroxidation, and activation of apoptotic pathways. For translation in the future, genetic validation experiments, in vivo models, and compound isolation are recommended.

## Data Availability

The datasets are available in the NCBI repository (https://www.ncbi.nlm.nih.gov) under accession numbers: SAMN42050866 (CK1), SAMN42050867 (CK2), SAMN42050868 (CK3), SAMN42050869 (T1), SAMN42050870 (T2), and SAMN42050871 (T3).
