# Supplementary material for: Sea buckthorn seed oil triggers mitochondrial dysfunction and apoptosis in Aspergillus niger by activating the AGE-RAGE-like signaling pathway
Source: Front Microbiol. 2026 Apr 21;17:1762938. doi: 10.3389/fmicb.2026.1762938 (PMC13139193; doi:10.3389/fmicb.2026.1762938)
Supplement: Supplementary file 1 [file Data_Sheet_1.DOCX]

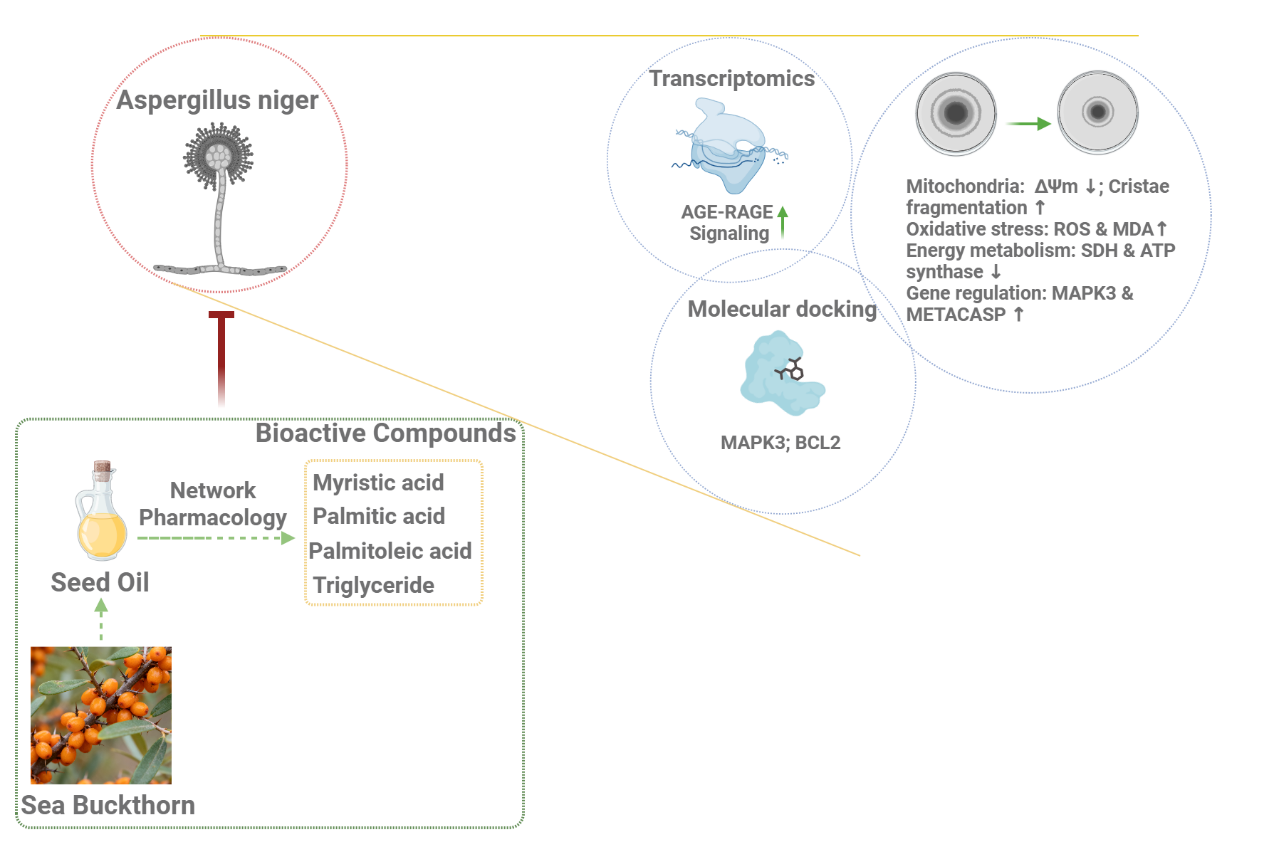


**Sea buckthorn seed oil triggers mitochondrial dysfunction and apoptosis in *Aspergillus niger* via the AGE-RAGE signaling pathway.**

An integrated approach combining network pharmacology and transcriptomics identified key bioactive compounds in sea buckthorn seed oil (including myristic acid, palmitic acid, palmitoleic acid, and triglycerides) and highlighted the crucial role of the AGE-RAGE signaling pathway. Molecular docking confirmed the stable binding of these compounds to core targets (MAPK3 and BCL2). Experimental validation demonstrated that the oil activates the AGE-RAGE pathway, leading to severe mitochondrial dysfunction characterized by loss of membrane potential (ΔΨm), cristae fragmentation, inhibition of succinate dehydrogenase (SDH) and ATP synthase activity, and a surge in oxidative stress markers (ROS and MDA). Concurrent upregulation of MAPK3 and the apoptotic executor METACASP drives the fungal cells into apoptosis, ultimately inhibiting *A. niger* growth.
